# Is there sufficient evidence to inform personal protective equipment choices for healthcare workers caring for patients with viral hemorrhagic fevers?

**DOI:** 10.14745/ccdr.v51i01a02

**Published:** 2025-01-02

**Authors:** Amanda Graham, Steven Ettles, Maureen McGrath, Toju Ogunremi, Jennifer Selkirk, Natalie Bruce

**Affiliations:** 1 Centre for Communicable Disease and Infection Control, Public Health Agency of Canada, Ottawa, ON

**Keywords:** personal protective equipment, PPE, healthcare worker, transmission, contamination, exposure, viral hemorrhagic fever, filovirus, Ebola

## Abstract

**Background:**

Ugandan health authorities declared an outbreak of Ebola disease (EBOD), caused by the Sudan virus, in September 2022. A rapid review was conducted to update the Public Health Agency of Canada's guidelines for infection prevention and control measures for EBOD in healthcare settings to prepare for potential introduction of cases.

**Objective:**

Summarize the available evidence on personal protective equipment (PPE) use by healthcare workers (HCWs) to prevent exposure to and transmission of viral hemorrhagic fevers (VHFs), including Ebola virus.

**Methods:**

Electronic databases were searched to identify peer-reviewed evidence published from July 2014–October 2022. Peer-reviewed primary studies and literature reviews, in English or French, reporting on PPE for VHFs and filoviruses in the healthcare context were eligible for inclusion. Literature review processes were conducted by two reviewers using DistillerSR® systematic review software and the Public Health Agency of Canada's Infection Prevention and Control Critical Appraisal Toolkit. An environmental scan of grey literature was also conducted to inform the rapid review.

**Results:**

The database search yielded 417 citations and 29 studies were considered eligible for critical appraisal. In total, 20 studies were included in the narrative synthesis of evidence. The evidence base was limited regarding comparative effectiveness of types of PPE for preventing exposure to and transmission of VHFs to HCWs. Four studies reported on exposure to and transmission of a VHF. Sixteen studies provided data on other relevant topics, such as simulated contamination and lab-based tests of PPE integrity.

**Conclusion:**

There is limited evidence with which to draw conclusions on the comparative effectiveness of PPE to prevent exposure to and transmission of VHFs to HCWs. Additional research is required to determine the optimal PPE to protect HCWs from exposure to and transmission of VHFs.

## Introduction

Viral hemorrhagic fevers (VHFs) are a group of diseases caused by enveloped, single-stranded RNA viruses belonging to six taxa, namely Filoviruses (i.e., Ebola and Marburg virus), Arenaviruses, Flaviviruses, Hantaviruses, Nairoviruses and Phenuiviruses ( ((1))). Ebola disease (EBOD) was first described in 1976 in two simultaneous outbreaks in two different countries, South Sudan and the Democratic Republic of Congo, and comprises six species, four of which are pathogenic to humans ( ((2,3))). Prior to 2014, a total of 2,387 cases had been recorded in localized rural African outbreaks, with an overall crude mortality of 67% ( ((1,2))). In 2014, an outbreak that occurred in West Africa, which lasted for two years, showed intense urban transmission and resulted in over 28,000 cases, with multiple countries including Italy, Mali, Nigeria, Senegal, Spain, the United Kingdom and the United States reporting imported cases ( ((4,5))).

Human-to-human transmission of Ebola virus (EBOV) occurs by direct contact (i.e., through non-intact skin or mucous membranes) with the blood or other body fluids (e.g., stool, emesis, urine, saliva, semen and sweat) of an infected individual and/or by indirect contact with environmental surfaces and fomites contaminated with infected blood or other body fluids ( ((1,2,4))). The risk of transmission increases with the amount of infectious material to which the individual is exposed ( ((5))). Investigations conducted to date have not identified human-to-human transmission of EBOV in the absence of direct contact with an infected case ( ((3,4))).

The use of effective personal protective equipment (PPE) for healthcare workers (HCWs) providing care to patients with suspected or confirmed VHF is essential to prevent HCW infection and nosocomial transmission. Close contact with confirmed or suspected cases without adequate infection prevention and control (IPC) precautions in place can result in HCW infection and mortality. In Sierra Leone, Guinea and Liberia alone, 513 deaths out of a total of 881 infected HCWs have been reported as of 2015 ( ((4))). Currently, there is little consensus on the safest PPE ensembles to protect HCWs from exposure to EBOV, with frequent jurisdictional inconsistency in PPE recommendations. Despite the gap in global consensus, Canadian IPC recommendations are developed with a precautionary approach, designed to prioritize the health and safety of HCWs.

In September 2022, the Ugandan health authorities declared another outbreak of EBOD, caused by Sudan EBOV species, which led to 142 confirmed cases, 22 probable cases, 55 confirmed deaths and 87 recovered patients ( ((2,6))). No cases were reported in Canada. Given this evolving epidemiological situation, a rapid review of the literature was conducted to inform the update of the Public Health Agency of Canada's (PHAC) guidelines for IPC measures for EBOD in healthcare settings. This review summarizes the available evidence on PPE use by HCWs to prevent exposure to and transmission of VHFs, including EBOV.

## Methods

### Literature search and eligibility criteria

Electronic search strategies were developed by the authors in consultation with a Health Canada Library librarian. Embase, MEDLINE, Global Health and Scopus databases were searched in October 2022. Studies published in either English or French from July 2014 to October 2022 were considered in this review.

Our research aimed to answer the following question: What literature exists related to PPE use by healthcare staff to prevent transmission of and exposure to viral haemorrhagic fevers? Search terms utilized covered a wide variety of healthcare settings, professions and PPE items. Search terms and population, exposure, intervention, control and outcomes (PICO) criteria for the literature search can be found in **Table 1**.

**Table 1:** Population, exposure, intervention, control and outcomes search criteria for systematic review

PICO criteria	Search criteria
Populations	Personnel: Dietician, food services, emergency medical technician (EMT), licensed practical nurse (LPN), medical radiation technologist, medical laboratory technologist (MLT), midwife, nurse practitioner (NP), paramedic, physician, physician assistant, registered nurse (RN), registered nurse assistant (RNA), registered practical nurse (RPN), respiratory therapist (RT), environmental services, cleaning staff, phlebotomist, porters, transportation workers Type of care: Acute care, hospital care, emergency care, critical care, intensive care, ambulatory care, out-patient care, community care, home care, respite care, palliative care, long-term care, complex continuing care, rehabilitation care, pre-hospital care, convalescent care, mental healthcare
Exposures	Viral haemorrhagic fever, filoviruses, Ebola, Lassa, Marburg, Crimean-Congo virus, Ebola virus disease (EVD), Sudan virus disease (SVD)
Interventions	Personal protective equipment (PPE), gowns, gloves, respirators, N95, powered air purifying respirators (PAPR), face protection, masks, visors, eye protection, goggles, glasses, aprons, Tyvek, coverall, boots, boot covers, shoe covers, hood
Comparison	Not relevant at this time
Outcomes	Exposure (event) Transmission (event), spread Contamination, self-contamination

Abbreviations: EMT, emergency medical technician; EVD, Ebola virus disease; LPN, licensed practical nurse; MLT, medical laboratory technologist; NP, nurse practitioner; PAPR, powered air purifying respirators; PICO, population, exposure, intervention, comparison and outcomes; PPE, personal protective equipment; RN, registered nurse; RNA, registered nurse assistant; RPN, registered practical nurse; RT, respiratory therapist; SVD, Sudan virus disease

### Study selection and data extraction

Duplicate studies were identified and removed. A screening tool was developed in Excel® for initial screening to verify that parameters for inclusion were met. Additional title and abstract screening to assess study design/format and relevance to use of PPE was conducted by two independent reviewers in DistillerSR®. Due to the breadth of results, the scope of studies eligible for inclusion was narrowed to include only those directly relevant to the use of PPE in relation to the outcomes of interest, based on the informed opinion of the reviewer. News articles, editorials, commentaries, opinion pieces, guidelines, policy statements, cost analyses and articles with legal/ethical foci were excluded. Additionally, research focusing on protocol development, as well as heat exhaustion and comfort during PPE use, were also excluded.

As the review was primarily meant to inform national IPC guidance, studies were restricted to those conducted in G20 countries with the addition of New Zealand at full-text review to ensure applicability to the Canadian context.

A full-text screening tool (DistillerSR) was developed to exclude studies based on the exclusions noted above, and to retrieve relevant details on study design, methodology and qualitative and quantitative results regarding relevant PPE. Full-text screening was conducted by two independent reviewers. Conflicts were resolved via consensus-building discussion between reviewers, with a third reviewer providing input if consensus could not be reached.

Included articles were critically appraised using PHAC's Infection Prevention and Control Guidelines Critical Appraisal Toolkit ( ((7))). This suite of tools is used to grade evidence in a systematic manner across several domains, including assessments of the study population and sampling methods, internal and external validity, ethics and control of confounding and bias. A summary result was assigned to the study based on strength of design, overall quality of the study and directness of evidence.

### Evidence synthesis

A narrative synthesis of the evidence was created to identify common study foci, areas of consensus and variation and gaps in the evidence base.

## Results

### Overview of included studies

**Figure 1:**
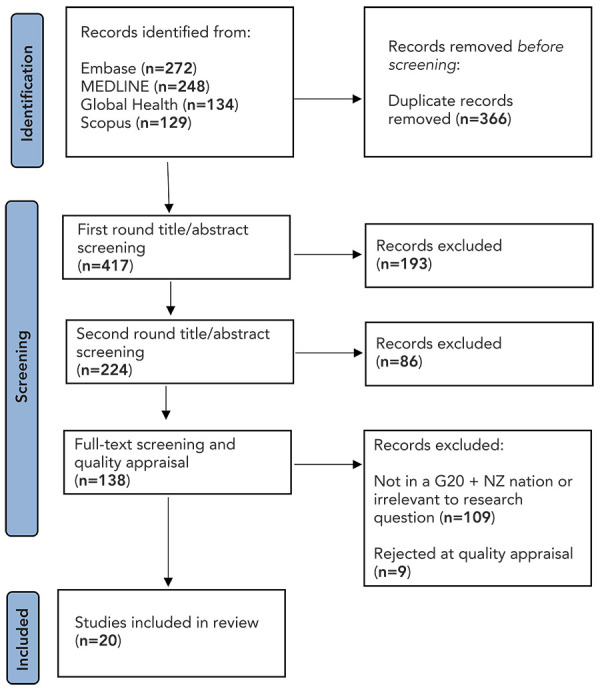
PRISMA flow-chart of literature review process Abbreviations: G20 + NZ, G20 countries plus New Zealand; PRISMA, Preferred Reporting Items for Systematic reviews and Meta-Analyses

Preferred Reporting Items for Systematic Reviews and Meta-Analyses (PRISMA) results can be found in **Figure 1**. Twenty studies were included in the analysis: eight laboratory studies, two randomized controlled trials, three non-randomized controlled trials, one cross-sectional study, four case reports and two literature reviews. A summary of the included evidence can be found as supplemental material in the **Appendix**.

### Contamination by fluorescence

Three studies ( ((8–10))) assessed contamination using fluorescent surrogate viruses, simulating enveloped and non-enveloped viruses. In each study, participants donned a complete ensemble of PPE commonly used for high-consequence infectious diseases (HCID). In two studies conducted by Casanova *et al.*, researchers primarily sought to measure detectable contamination of PPE and of individuals, indicated by fluorescent markings ( ((8,9))). In both studies, there was no detection of the enveloped surrogate virus after doffing PPE, but several instances of contamination by non-enveloped virus, particularly on participants' inner gloves. Detection of contamination, on the inner gloves but not on the hands, suggested that hand hygiene steps in doffing processes were protective against self-contamination with enveloped and non-enveloped viruses.

The study by Mumma *et al.* ( ((10))), conducted failure modes and effects analysis and fault tree analysis to identify and quantify the risk of errors in a doffing protocol. While participants completed simulated patient care activities and doffed Ebola-level PPE, risks of failure modes were identified and quantified. The extent to which errors contributed to self-contamination were delineated. Three groups of failure modes were found to have higher risk indices, characterized by their frequency and severity. These included hand hygiene-related errors, compromised PPE (especially at hands and wrists) and mishandling of PPE (especially the powered air purifying respirator [PAPR] hood and face shield). A subsequent study conducted by Mumma *et al.* ( ((11))) used similar methodology to identify potential errors in a PPE doffing process and their frequency. Corroborating the results of the previous study, hand hygiene and PAPR hood removal were associated with greater risk of error. Removal of the outermost garment and of boot covers also showed above-average risk of error in the PPE doffing process.

### Comparing personal protective equipment ensembles

Chughtai *et al.* ( ((12))) tested ten established PPE protocols for EBOD. The rate of self-contamination among participants was lower for PPE protocols using gowns compared to protocols using coveralls. Powered air purifying respirators were observed to be more protective compared to N95 respirators, potentially due to lower risk of self-contamination with fewer items of PPE and the incorporation of assisted doffing.

A study by Suen *et al.* ( ((13))), which compared the efficacy of three PPE ensembles, reported some differences in contamination across the ensembles. Standard Ebola PPE (PPE1), which included neck-to-ankle coveralls, a water-resistant gown, double nitrile gloves and a hood, was compared to another Ebola PPE set (PPE2), which included front-zip coveralls, a plastic apron and double gloves. Both PPE1 and PPE2 used boots, a face shield and an N95 respirator, and were compared against a reference PPE ensemble used for routine practices and aerosol-generating procedures (PPE3). The study found less frequent contamination of participants' clothing in small patches during removal of PPE1 (median of 5.00 small patches of contamination), compared to PPE2 and PPE3 (median 7.00 patches). Additionally, less contamination overall for Ebola-specific PPE ensembles (PPE1, PPE2) was found compared to the reference PPE (PPE3).

Hall *et al*. ( ((14))) compared a basic PPE ensemble (surgical mask, standard length apron, one pair short gloves, own footwear) against established PPE protocols in use across HCID units in the United Kingdom. Across protocols, 1,584 contamination events were recorded after the completion of a simulation exercise, and twelve contamination events post-doffing. Identified breaches were related to protocol failure or complications in the doffing processes. In a follow-up study, Poller *et al*. ( ((15))) replicated the testing using a new PPE ensemble for HCIDs developed by an expert working group. While frequency of post-simulation contamination events was comparable to those observed by Hall *et al*., no residual contamination was observed post-doffing with the new PPE ensemble. Notable features of the ensemble included use of a FFP3 respirator, anti-infection transfer hood, full face visor, rear-fastening reinforced and fluid-resistant surgical gown, wide and extra-long medium thickness plastic apron, three layers of gloves and surgical wellington boots.

A randomized controlled trial ( ((16))) utilized a single Ebola PPE ensemble but compared two different training packages to assess fluorescent contamination in each group after undergoing simulated contamination. Both groups received basic IPC and PPE training. The intervention arm received considerably more teamwork-focused training, including strategies, defined roles and responsibilities and a demonstration of the doffing process. Upon examination, self-contamination was observed to be significantly lower in the intervention group compared to controls.

### Assessing personal protective equipment integrity

Gao *et al.* ( ((17))) tested thirteen brands of nitrile (n=8) and latex (n=5) gloves, examining tensile strength and ultimate elongation without (control) and with one to six applications of alcohol-based hand rub (ABHR). Overall, ethanol based ABHR had little to no effect on elongation of most gloves but resulted in decreased tensile strength for some nitrile gloves after up to six applications. Despite this, all but two relatively thin nitrile gloves continued to meet National Fire Protection Association standards for tension strength and elongation.

Nikiforuk *et al.* ( ((18))) measured environmental persistence of EBOV and viral RNA on various PPE materials. Ebola virus remained viable on all materials from 24–72 hours post-inoculation, except for on gloves (less than an hour) and goggles (less than 24 hours). Ebola virus penetration through PPE materials was measured using dry and phosphate-buffered saline-saturated samples of a hood, coveralls and respirator. Saturated samples were found to provide less protection compared to dry material. Similar results were observed when dry and saturated samples of a surgical mask and two respirators were tested. Overall, saturated PPE materials provided less protection compared to dry samples, with penetration of EBOV in seven of 21 saturated samples compared to one of 21 dry samples of the same materials.

Jaques *et al*. ( ((19))) conducted Elbow Lean Tests, using various levels of pressure, on isolation gowns and coveralls to measure resistance of the continuous and discontinuous regions of garments to penetration of simulated bodily fluids. Overall, higher pressure led to higher failure rates across all types of garments for both continuous and discontinuous regions. In discontinuous regions, coveralls that had high failure rates in seam regions and zippers were not protective, but heat-sealed seams performed better. Only one garment model, a gown, demonstrated nearly 100% barrier protection for the whole garment, with one failure out of 42 tests.

### Case reports

Four case reports were included, describing IPC measures taken for the care of patients under investigation, or confirmed to have EBOD or other VHFs ( ((20–23))). Reporting of specific PPE items and/or ensembles used in patient care was limited and few details were provided on the effectiveness of PPE in preventing exposure and transmission. Despite their limitations, these studies could not be reasonably excluded based on the established inclusion criteria and were therefore included in the analysis.

### Literature reviews

Two literature reviews were included in the analysis. Hersi *et al*. ( ((24))) assessed the benefits and harms of Ebola-specific PPE compared to potentially less robust PPE in the context of HCWs caring for patients with filoviruses. Despite comprehensive methodology, insufficient evidence was available to draw conclusions on the effectiveness and potential harms of robust PPE compared to the alternative.

Licina *et al*. ( ((25))) evaluated the effect of PAPRs for respiratory protection against highly virulent infectious diseases, including EBOD, compared to other devices such as N95/FFP2 respirators, on HCW infection rates and contamination. Equivalent rates of infection were demonstrated in cohorts using PAPRs compared to other appropriate respiratory protection. The review did identify some low-quality evidence pointing to the advantages of PAPRs, compared to alternative respiratory protection, for wearer protection from cross-contamination and in doffing simulation studies.

### Quality appraisals

Twenty-nine studies underwent quality appraisal using PHAC's Critical Appraisal Toolkit ( ((7))). Nine studies were rejected for further inclusion at this stage due to a lack of relevance to our research question. Of the 20 included studies, 2 studies were appraised as being of high quality ( ((17,18))), 14 as being of medium quality and 4 as being low quality. For all studies, the directness of evidence was determined to be extrapolation, as results were related to a different research question, or were investigated under artificial conditions. Full quality appraisal results can be found in **Table 2**.

**Table 2:** Quality appraisal results of included studies

Study (reference)	Study design	Strength of design^a^	Directness of evidence	Overall quality of study
Andonian *et al.*, 2019 ( ((16)))	RCT	Strong	Extrapolation	Medium
Bell *et al.*, 2022 ( ((26)))	RCT	Strong	Extrapolation	Low
Barratt *et al*., 2015 ( ((20)))	Case report	Weak	Extrapolation	Medium
Casanova *et al.*, 2018 ( ((8)))	Laboratory study	Strong	Extrapolation	Medium
Casanova *et al.*, 2016 ( ((9)))	NRCT	Strong	Extrapolation	Low
Chughtai *et al.*, 2018 ( ((12)))	Laboratory study	Strong	Extrapolation	Medium
Cummings *et al.*, 2016 ( ((21)))	Case report	Weak	Extrapolation	Low
Drew *et al.*, 2016 ( ((27)))	NRCT	Strong	Extrapolation	Medium
Gao *et al.*, 2016 ( ((17)))	Laboratory study	Strong	Extrapolation	High
Hall *et al.*, 2018 ( ((14)))	NRCT	Strong	Extrapolation	Low
Haverkort *et al.*, 2016 ( ((22)))	Case report	Weak	Extrapolation	Medium
Hersi *et al.*, 2015 ( ((24)))	Literature review	Not applicable^b^	Extrapolation	Medium
Jaques *et al.*, 2016 ( ((19)))	Laboratory study	Strong	Extrapolation	Medium
Lehmann *et al.*, 2017 ( ((23)))	Case report	Weak	Extrapolation	Medium
Licina *et al.*, 2020 ( ((25)))	Literature review	Not applicable^b^	Extrapolation	Medium
Mumma *et al.*, 2019 ( ((11)))	Cross-sectional study	Weak	Extrapolation	Medium
Mumma *et al.*, 2018 ( ((10)))	Laboratory study	Strong	Extrapolation	Medium
Nikiforuk *et al.*, 2017 ( ((18)))	Laboratory study	Strong	Extrapolation	High
Poller *et al.*, 2018 ( ((15)))	Laboratory study	Strong	Extrapolation	Medium
Suen *et al.*, 2018 ( ((13)))	Laboratory study	Strong	Extrapolation	Medium

Abbreviations: NRCT, non-randomized controlled trial; RCT, randomized controlled trial

^a^ Strength of study design is determined based on rankings assigned in the Public Health Agency of Canada's Critical Appraisal Toolkit

^b^ Literature reviews without meta-analyses are not assessed on study design. Quality of review methods used is captured under “overall quality of study”

## Discussion

The review identified 20 relevant studies addressing PPE use in the context of VHFs, such as EBOD, in high-income country contexts. A quality appraisal of evidence was completed with most studies ( ((19))) ranking low-to-medium quality; unfortunately, the lower quality of many studies limited our ability to extrapolate to real world scenarios. We concluded that there was insufficient evidence to draw conclusions on the comparative effectiveness of PPE to prevent exposure to and transmission of VHFs, including EBOV, to HCWs. Given this gap in evidence on PPE effectiveness, the determination of PPE ensembles for EBOD should consider a precautionary approach.

Nine studies with varying methodologies involved detecting contamination by fluorescent markers. Two studies showed that use of HCID PPE resulted in no self-contamination, suggesting the ensembles were effective. Further, studies comparing EBOD/HCID-specific PPE ensembles against basic or routine practice PPE consistently showed that more robust PPE ensembles are significantly more protective against contamination ( ((13–15))). However, comparing efficacy of enhanced PPE combinations proves difficult due to heterogeneity of the ensembles compared between studies, methods used to simulate and record contamination, differences in reporting of contamination and variations in training and experience among participants. Due to the varied scope and methodology of the independent studies that met the inclusion criteria, a meta-analysis that would quantitatively summarize overall findings of the results was not possible. As such, it is difficult to draw conclusions on the protective effects of individual items or combinations of PPE. Most ensembles varied significantly with respect to body protection, gloving, head coverage and footwear. Other aspects of protocols (when reported) also varied, including hand hygiene and doffing assistance.

Three medium-to high-quality ( ((17–19))) laboratory studies using differing methodologies assessed the integrity of PPE items, examining degradation of PPE materials and penetration of EBOV, surrogate virus or simulated bodily fluids through samples. Personal protective equipment tested included gloves ( ((17,18))), gowns and coveralls ( ((18,19))) and surgical masks and respirators ( ((18))). These studies found that the PPE studied was generally resistant to ABHR degradation and that moisture-saturated PPE materials tended to provide less protection than dry materials, indicating the importance of avoiding body fluid contamination and excessive sweating while donned. Further work should be done to elucidate the comparative performance of other models of PPE to determine which provide the greatest protection in the event of moisture saturation and activity-induced pressure.

Despite a well-designed and comprehensive review methodology, Hersi *et al.* ( ((24))) were not able to reach a conclusion on the effectiveness of various forms of PPE for HCWs providing care to patients with VHFs, owing to similar issues with the body of evidence, such as low study quality and heterogeneity among PPE components and reporting. This result aligns with our findings.

When examining the role of PAPRs ( ((25))), investigators were unable to find differences in protection compared to other forms of respiratory protection. This result is inconsistent with the work by Chughtai *et al.* ( ((12))), which showed that PAPRs were observed to be more protective compared to N95 respirators. Though there were a number of limitations in the work by Chughtai *et al*., further work is needed to determine the true safety of PAPR use, and its role in self-contamination, when caring for patients with VHFs.

Another issue that was noted was PPE reporting across studies was often inconsistent, with a number of studies citing PPE ensembles only as “high consequence” or “Ebola-specific” PPE, making it difficult to determine individual components and their comparability to PPE used in other studies. Additionally, steps for donning and doffing often were not reported, posing another barrier to compare results.

### Strengths and limitations

One strength of this review was that it utilized a standardized screening and data extraction form within a reference management software, which helped to reduce bias and ensure data integrity via consistent collection and reporting. Results were also critically appraised to assess domains of bias, with some studies being excluded when methodology was deemed to be too poor. Another strength of this review was that search criteria excluded countries outside of the G20 and New Zealand, to ensure compatibility of findings with Canadian healthcare settings when drafting IPC practice recommendations. Further, the addition of multiple focus criteria during the screening process facilitated selection of studies that were more comparable and more relevant to the research question, thereby enhancing our analysis.

One weakness of this study is the limited number of studies included and the overall heterogeneity among the methodology and outcomes. Most studies were also limited by small sample sizes (fewer than 20 participants). Given these issues and the inability to conduct further statistical analysis, it was difficult to draw firm conclusions from the available data, impacting our ability to reach evidence-based conclusions.

### Conclusion and future directions

To our knowledge, this is the first literature review conducted in Canada to address the research question: What literature exists related to PPE use by healthcare staff to prevent transmission of and exposure to viral haemorrhagic fevers in high-income contexts? This summary provides important insight into the state of knowledge of this topic as well as identifying areas needing further exploration.

Overall, there was limited evidence to draw conclusions on the comparative effectiveness of PPE to prevent exposure to and transmission of VHFs to HCWs. The current body of evidence would benefit from a more robust comparison of different PPE components and models, using standardized methods for data collection and reporting to ensure comparability amongst studies. There is a notable gap in strong study designs (such as randomized controlled trials and studies that involve large numbers of participants), which would produce more robust results and allow for statistical analysis and modelling. If feasible, conducting more studies during outbreaks of VHFs, or during actual care of EBOD patients, would provide greater insight into outcomes encountered in the real world compared to simulation studies.

## Appendix

Supplemental material is available upon request to the author: steven.ettles@phac-aspc.gc.ca
